# Differential diagnosis of primary intrapulmonary thymoma: a report of two cases

**DOI:** 10.1186/s40792-015-0061-1

**Published:** 2015-07-08

**Authors:** Fumihiro Ishibashi, Yasumitsu Moriya, Hajime Tamura, Yukiko Matsui, Toshihiko Iizasa

**Affiliations:** Division of Thoracic Diseases, Chiba Cancer Center, 666-2, Nitona-cho, Chuo-ku, Chiba 260-8717 Japan

**Keywords:** Thymoma, Lung, Surgery, Pathology

## Abstract

Primary intrapulmonary thymomas (PITs), which are intrapulmonary tumors without an associated mediastinal component, are very rare. The diagnosis of a PIT can be difficult. Here, we report two cases of resected PITs that were difficult to differentiate from other lung tumors. The patients, of a 62-year-old man and a 64-year-old woman, had no significant symptoms and were both referred to our hospital due to the presence of an abnormal shadow on chest computed tomography (CT). The patients underwent ^18^F-fluorodeoxyglucose positron emission tomography-CT (FDG-PET/CT) and subsequently tumor excision. A PIT was confirmed histopathologically in the surgical specimens from both patients. In one case, the tumor consisted of a type A thymoma without abnormal FDG uptake. In the other case, the tumor consisted of a type B2 thymoma presenting with weak FDG uptake. This report thus documents two cases of PITs with different histopathologic and FDG-PET/CT findings. Thoracoscopic surgery is essential in the differential diagnosis between PITs and other lung tumors.

## Background

Thymomas are tumors derived from thymic epithelial cells, reported to have an incidence of 0.15 per 100,000 person-years [1]. Primary intrapulmonary thymomas (PITs) are very uncommon and have been regarded as a disease of unknown etiology. They are defined as tumors below the visceral pleura or entirely circumscribed by lung parenchyma without evidence of a thymic lesion in the anterosuperior mediastinum, which display the characteristic histological features of thymomas [2]. PITs are difficult to diagnose. Furthermore, to the best of our knowledge, there are no reports on the use of ^18^F-fluorodeoxyglucose positron emission tomography (FDG-PET) to evaluate patients with PIT. Herein, we report two patients with PIT, in whom a low-grade malignant pulmonary tumor or metastasis was suspected on diagnostic imaging, but PIT was ultimately diagnosed based on tumor specimens excised via video-assisted thoracoscopic surgery (VATS).

## Case presentation

### Case 1

**Fig. 1:**
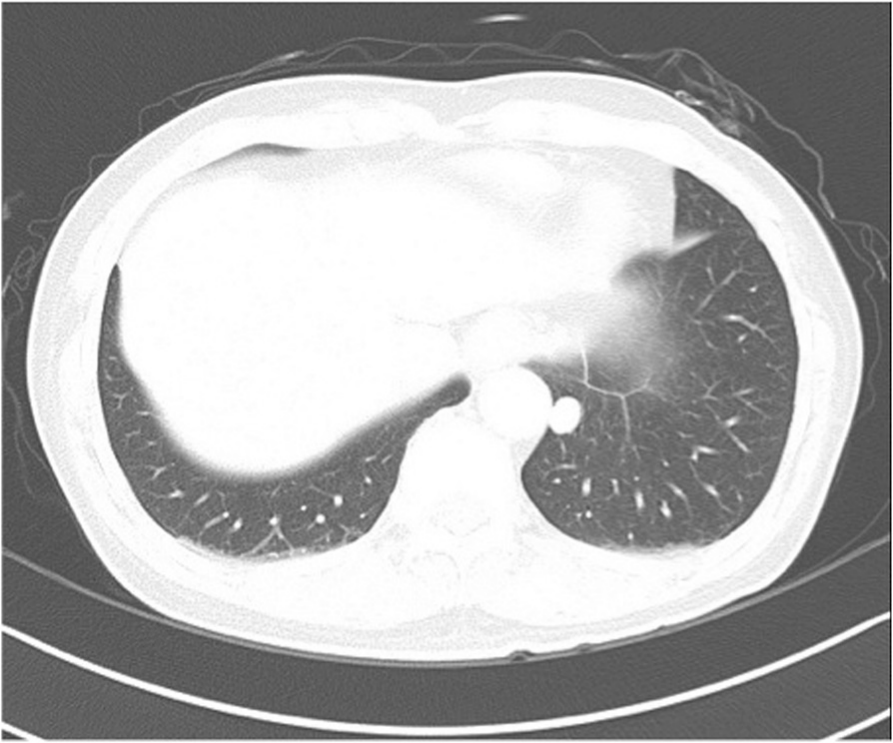
Chest computed tomography (CT) of case 1 shows a nodular shadow in the left S10

**Fig. 2:**
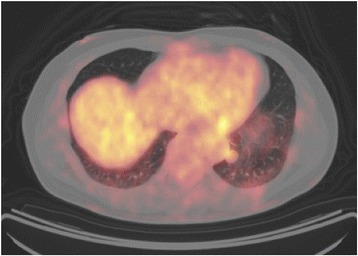
^18^F-fluorodeoxyglucose positron emission tomography (FDG-PET) revealed a nodular lesion with no abnormal uptake of the tracer

A 62-year-old man was referred to our department for evaluation of an abnormal shadow on chest computed tomography (CT). A solitary, very small, round opacity in the left lung was incidentally detected on chest CT during a routine medical examination 2 years previously. The lesion was followed radiologically at regular intervals, and the size of the nodule had increased slightly. Chest CT revealed a 15 × 10 mm tumor in the left S10 (Fig. 1). No other intrathoracic abnormalities were found, and there were no solid lesions in the anterior mediastinum. FDG-PET/CT revealed a nodular lesion without abnormal uptake of tracer (Fig. 2). Tumor marker levels, including carcinoembryonic antigen (CEA), cancer antigen (CA)19-9, squamous cell carcinoma antigen (SCC), and progastrin-releasing peptide (Pro-GRP), were all within normal limits. We suspected the tumor to be a carcinoid or hamartoma, and a wedge resection of the tumor was performed under VATS. Intraoperative examination of a frozen specimen revealed a spindle cell tumor, but no malignant cells were detected.

**Fig. 3:**
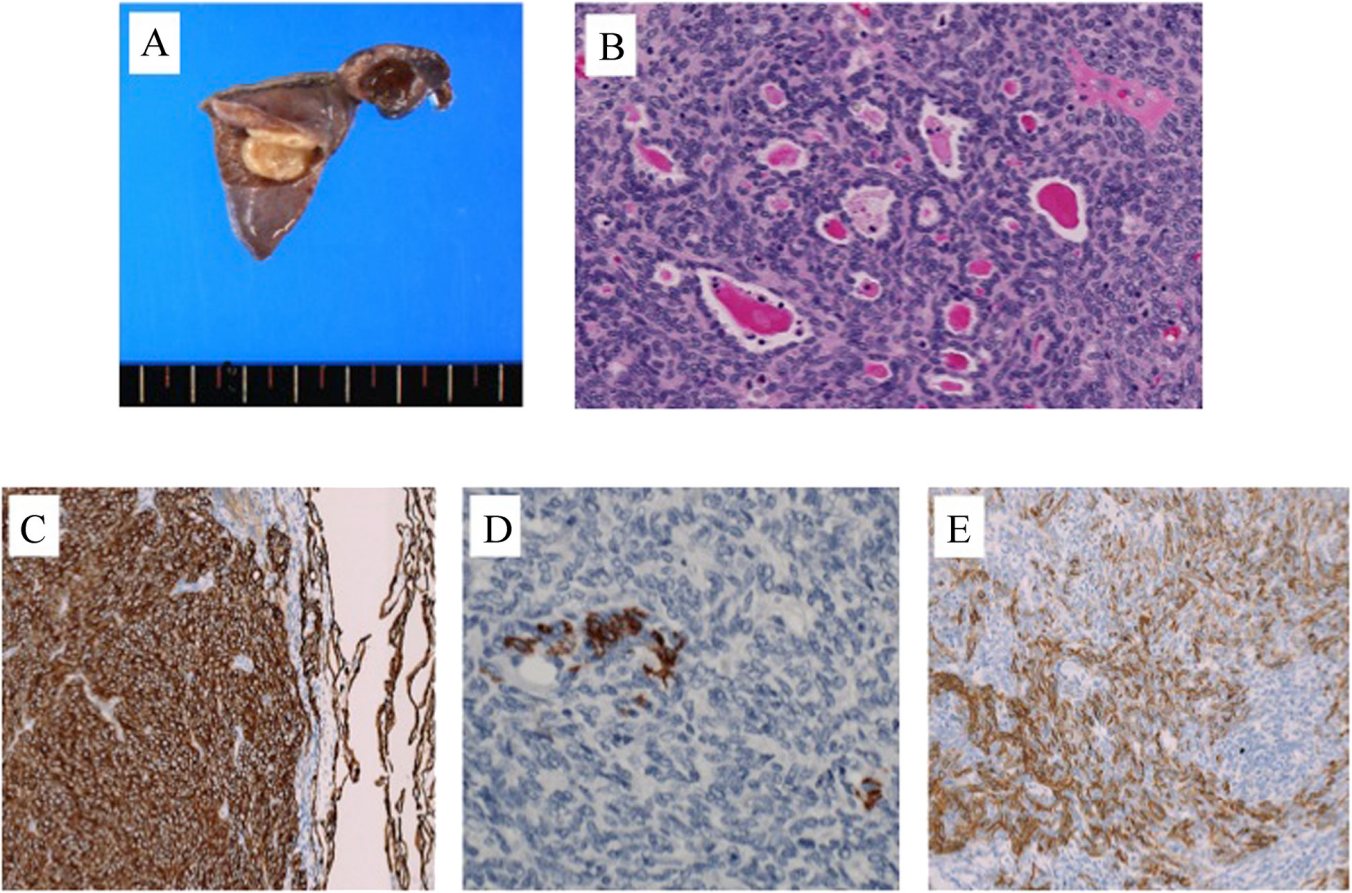
The cut surface of the resected specimen of case 1 shows a well-circumscribed and whitish nodule within the lung (**a**). Microscopic findings of the tumor include dense proliferation of spindle cells with a minority arranged in pseudoglands and a cystic lesion. An accumulation of lymphocytes was also observed within the tumor (H&E stain) (**b**). The tumor cells were diffusely positive for AE1/AE3 (**c**), and the lymphocytes were focally positive staining for CD1a (**d**) and CD20 (**e**)

Macroscopic examination found a white, well-circumscribed nodule within the lung that did not involve the visceral pleura (Fig. 3a). Histopathologic evaluation determined that there was a clear boundary between the tumor and lung tissue. The tumor consisted of a dense proliferation of spindle cells, with a few spindle cells making up pseudoglandular structures, and a cystic lesion. A small cluster of infiltrating lymphocytes was also observed within the tumor (Fig. 3b). Immunohistochemical studies revealed that the tumor cells were diffusely positive for cytokeratin AE1/AE3 and cluster of differentiation (CD)99 antigen and focally positive for epithelial membrane antigen (EMA); the lymphocytes were positive for CD1a and terminal deoxynucleotidyl transferase (TdT) and CD20 (Fig. 3c–e). According to the World Health Organization (WHO) classification, these histologic findings were consistent with type A thymoma [3].

### Case 2

**Fig. 4:**
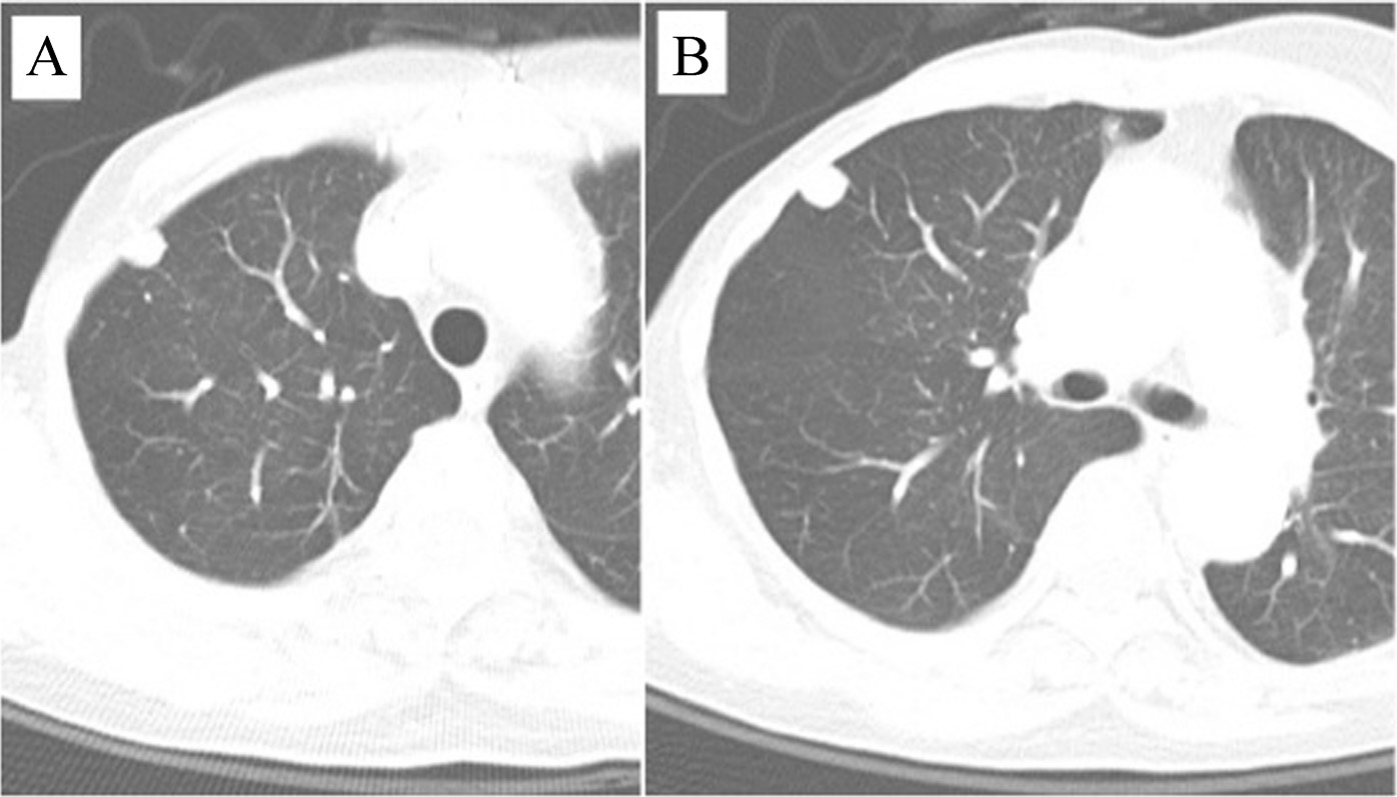
Chest CT of case 2 shows a nodular shadow in the right S1 (**a**) and in the right S3 (**b**)

**Fig. 5:**
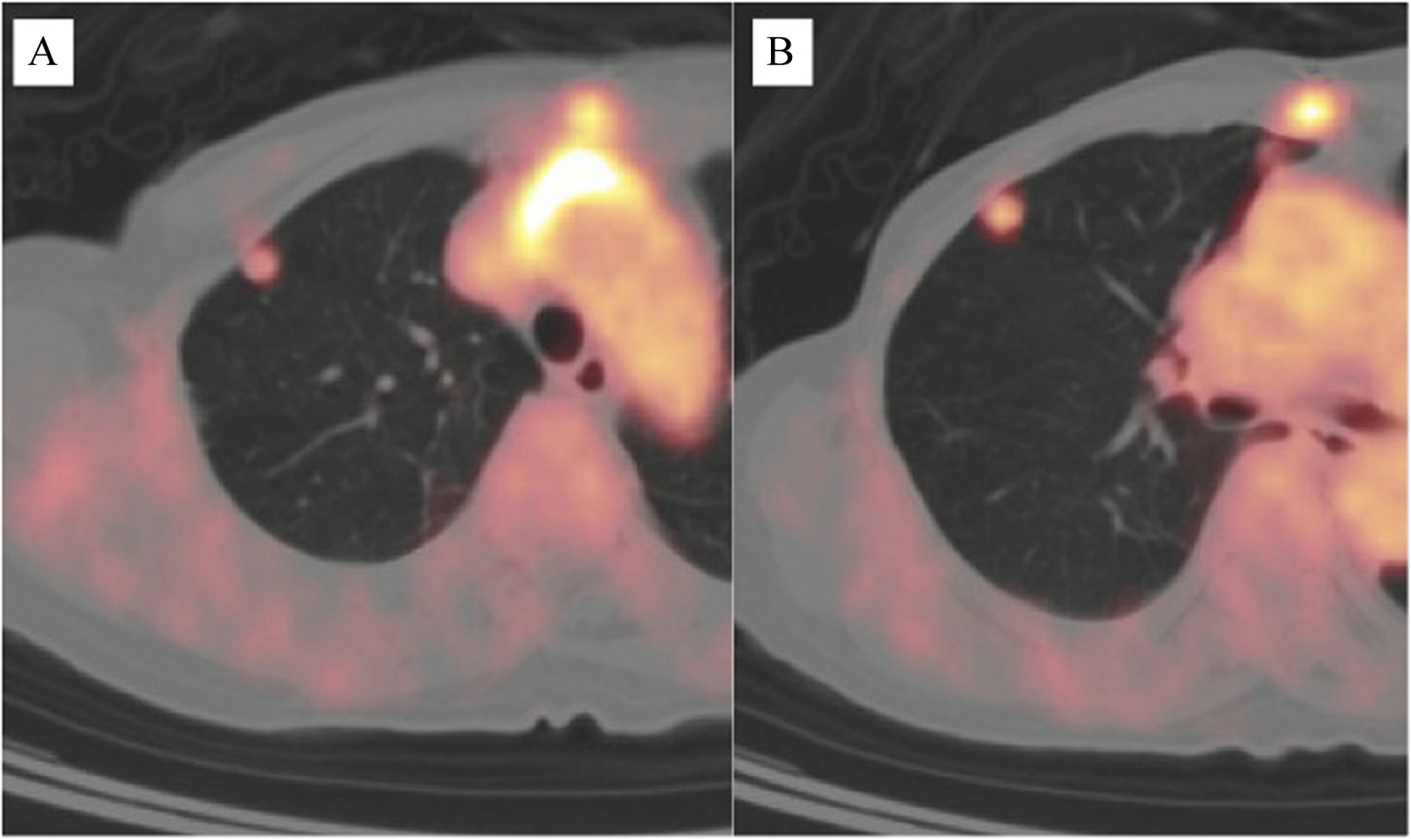
FDG-PET showed abnormal accumulation in the right S1 tumor (SUV max 2.35) (**a**) and in the right S3 tumor (SUV max 3.45) (**b**)

A 64-year-old woman was noted to have an abnormal lesion on chest radiography. She had undergone a total mastectomy for cancer of the right breast 26 years previously and replacement of the ascending aorta for aortic dissection (via median sternotomy) 3 years previously. Chest CT revealed a 9-mm tumor in the right S1 and an 11-mm tumor in the right S3 (Fig. 4a, b). There were no solid lesions in the anterior mediastinum. FDG-PET/CT showed slight uptake (maximum standardized uptake value [SUV max] 2.35 and 3.45, respectively) by the two nodular lesions (Fig. 5a, b). Tumor marker levels were normal, except for slightly elevated SCC. Because pulmonary metastases from breast cancer were suspected, we performed a wedge resection under VATS.

**Fig. 6:**
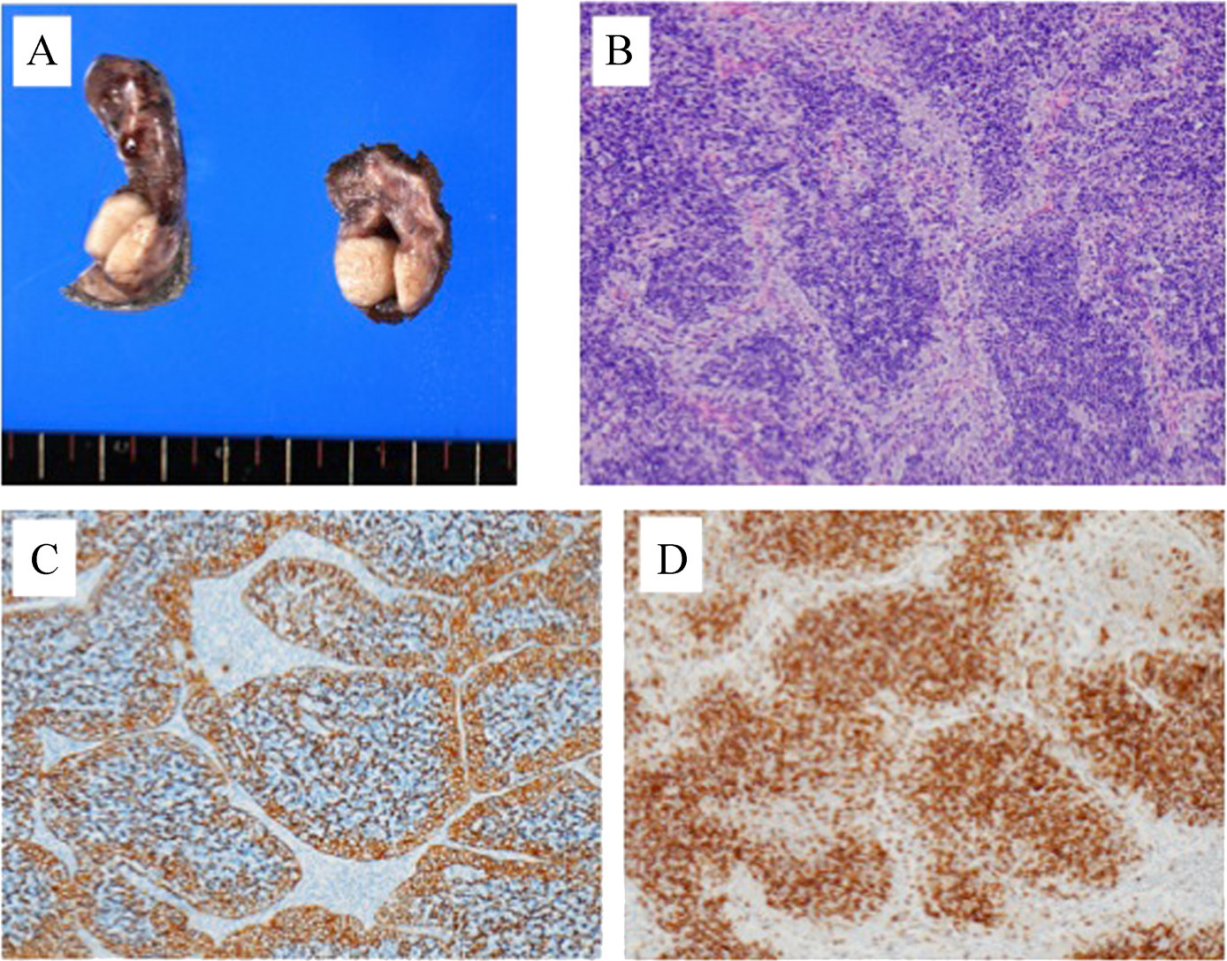
The cut surface of the resected specimen of case 2 shows S1 (*left*) and S3 (*right*) with well-circumscribed and whitish nodules within the lung (**a**). Microscopic findings of the S1 tumor show lobulated lesions with vascular septa. Each lobule consists of epithelial cells palisading along the septa and centrally located small lymphocytes (H&E stain) (**b**). The epithelial cells stain positive for AE1/AE3 (**c**), and the lymphocytes stain positive staining for CD1a (**d**)

Macroscopic examination found well-circumscribed, white nodules (Fig. 6a). Histopathologic evaluation of the S1 nodule found lobulated lesions with vascular septa. Each lobule consisted of epithelial cells palisading along the septa and centrally located small lymphocytes (Fig. 6b). Immunohistochemical evaluation revealed that the epithelial cells were positive for AE1/AE3 (Fig. 6c), and the lymphocytes were positive for CD1a and TdT (Fig. 6d). According to the WHO classification, these histologic findings were consistent with type B2 thymoma [3].

## Discussion

Primary intrapulmonary thymomas (PITs) are uncommon lung tumors. To the best of our knowledge, 32 cases of PITs have been reported in the English and Japanese literature since 1951, not counting our cases [2, 4–11].

Here, we present a summary of the clinicopathologic features of all 34 cases. The patients’ ages ranged from 14 to 79 years, and half the patients were females. The site of occurrence was the right pulmonary lobe in 21 cases, the left lobe in 12 cases, unknown in 1 case, the upper lobe in 22 cases, and the lower lobe in 11 cases. Three cases, including our second case 2, were multifocal within the same lung. The sizes of the lesions ranged from 9 to 128 mm in diameter. The presenting symptoms of PITs have included chest pain, cough, and hemoptysis, which also occur in patients with lung cancer, but most lesions were discovered incidentally on radiographic examination in asymptomatic individuals. Based on the WHO classification [3], there were 7 PIT type A cases, 8 type AB, 6 type B1, 5 type B2, and 7 type B3; 1 case was not classified. There were 3 patients with PIT and myasthenia gravis, and 2 patients with PIT and immunodeficiency, which is known as Good syndrome.

PIT appears on CT as a well-circumscribed, heterogeneous mass, such as seen in our patients. The differential radiologic diagnosis of PIT includes low-grade malignant tumor, metastatic lung tumor, hamartoma, sclerosing hemangioma, lipoma, and angiolipoma. Performing a preoperative diagnosis is difficult, and most PITs are diagnosed postoperatively [10].

Histological features of the tumor showed that the lesion was composed of dense proliferation of spindle cells with a minority arranged in pseudoglands and a cystic lesion in case 1, and there were lobulated lesions with vascular septa, consisted of epithelial cells palisading along the septa and centrally located small lymphocytes in case 2. The differential diagnosis of PIT includes a wide range lymphoma and primary or metastatic carcinoma of the lung. Immunohistochemical staining is helpful in this differentiation. Epithelial cells are stained by AE1/AE3 or EMA, and lymphocytes in thymomas are stained by T cell phenotypes [12]. Epithelial cell-dominant thymoma shares the same immunoreactivity for AE1/AE3 or EMA as metastatic carcinoma or poorly differentiated carcinomas of the lung, but PIT appeared as well-circumscribed lesions, with low mitotic activity and minimal atypia. The antigen CD20 is also helpful for diagnosis of type A thymoma because CD20-positive B cells are found if focal micronodular areas with a lymphoid stroma is present [3]. In our case, these tumors were diagnosed type A thymoma and type B2 thymoma in the WHO classification, respectively. Immunohistochemical staining combined with gross and microscopic pattern of the tumor and patient’s clinical history is helpful in differentiating a PIT from any other intrathoracic neoplasm [10].

FDG-PET can be applied to the diagnosis of malignant neoplasms because it is designed to detect and assess the degree of carbohydrate metabolism in malignant tissue [13]. The PITs of our two patients had different histopathologic manifestations and FDG-PET findings. The SUVmax values of the lesions of case 2 were markedly higher than the value of the lesion of case 1. It has been reported that FDG accumulation was highly correlated with WHO histologic subtypes of thymic epithelial tumors [14, 15]. Notably, the degree of FDG uptake has been positively correlated with the grade of malignancy of PITs as with the case of thymic epithelial tumors.

Most case reports of PIT have described the tumors as slow-growing, indolent, and asymptomatic until large enough to cause localized effects such as pain and bronchial obstruction [2]. The best therapy for PIT is complete resection, which for these tumors leads to good outcomes [6, 8, 10]. In their systematic review of 25 cases of PIT, Myers et al. [2] reported that the survival of surgically managed patients was significantly better than the survival of conservatively managed patients (log-rank test, *P* = 0.039). Histologic subtype (WHO classification) and tumor size did not significantly affect survival. Myasthenia gravis and the Good syndrome significantly decreased survival (log-rank test, *P* = 0.039). Because complete surgical resection was obtained for both of our cases, we believe that their postoperative outlook is good.

## Conclusions

PITs are very rare lesions that are difficult to diagnose. This report described two cases of PITs, which manifested different histopathologic and FDG-PET findings. If complete surgical resection of the tumor is possible, the postsurgical outlook is good.

## Consent

Written informed consent was obtained from the patients for publication of this case report and any accompanying images. A copy of written consent is available for review by the Editor-in-Chief of this journal.
